# Antibiotic resistance and molecular characterization of non-invasive clinical *Haemophilus influenzae* isolates in Germany 2019 and 2020

**DOI:** 10.1093/jacamr/dlae197

**Published:** 2024-12-10

**Authors:** Thiemo Frank, Esther Wohlfarth, Heike Claus, Manuel Krone, Thiên-Trí Lâm, Michael Kresken, Study Group ‘Antimicrobial Resistance’ of the Paul Ehrlich Society for Infection Therapy, L Artz, B Baadte, U Betz, J Cremer, U Eigner, R Geisel, C Haas, J Esser, I Fenner, R Ferner, Y Grundmann, I Hamann, T Hermann, C Friederichs, I Haftendorn, I Hoffmann, W M Holfelder, W Hönerlage, F Hugo, M Kolbert, S Krämer, R Krajewski, E Kühnen, D Mack, A Mair, A Meerbach, A Pranada, M Prian, I Purr, A Reinecke, B Reinhardt, H Sahly, S Schmitt, U Schuhmacher, A Siedlaczek, G Sitaru, S Sperber, H Wisplinghoff, D Wolff, S Wydra, C Zimmer, Sabrina Hebling, G Anlauf, E Berwian, M Korkmaz, S Wernicke

**Affiliations:** Institute for Hygiene and Microbiology, National Reference Center for Meningococci and Haemophilus influenzae, University of Würzburg, Würzburg, Germany; Antiinfectives Intelligence GmbH, Cologne, Germany; Institute for Hygiene and Microbiology, National Reference Center for Meningococci and Haemophilus influenzae, University of Würzburg, Würzburg, Germany; Institute for Hygiene and Microbiology, National Reference Center for Meningococci and Haemophilus influenzae, University of Würzburg, Würzburg, Germany; Institute for Hygiene and Microbiology, National Reference Center for Meningococci and Haemophilus influenzae, University of Würzburg, Würzburg, Germany; Antiinfectives Intelligence GmbH, Cologne, Germany

## Abstract

**Background:**

*Haemophilus influenzae* (Hi) is known as a cause of invasive and non-invasive diseases. Especially ear, nose and throat (ENT) infections are common reasons for antibiotic prescriptions in outpatient settings in Germany. Therefore, antibiotic resistance surveillance is important to provide the basis of recommendations for the empirical usage of antibiotic agents.

**Objectives:**

To provide data on susceptibility rates of oral antibiotics for non-invasive clinical Hi isolates in Germany and to investigate molecular resistance patterns of β-lactams, ciprofloxacin, doxycycline and trimethoprim/sulfamethoxazole.

**Methods:**

Isolates were collected from a sentinel network of diagnostic laboratories in a prospective multicentre prevalence study. Antibiotic susceptibility testing was done with a commercial broth microdilution kit. MICs were interpreted according to EUCAST guidelines. Resistance gene sequencing and WGS were performed to analyze molecular antibiotic resistance patterns and genetic relationships between the isolates.

**Results:**

In total, 215 Hi isolates were collected from 23 laboratories across Germany. The highest resistance rates were found for amoxicillin (*n* = 30; 14%), cefuroxime (*n* = 40; 18.6%) and trimethoprim/sulfamethoxazole (co-trimoxazole) (*n* = 34; 15.8%). Resistance to amoxicillin was mainly due to *bla_TEM-1_* (*n* = 29; 96.7%). PBP3 alterations were found in 39 of 40 cefuroxime-resistant isolates (97.5%). Two of the cefuroxime-resistant isolates harboured PBP3 mutation patterns that have not yet been associated with cefuroxime resistance; in one of them, a known *lpoA* mutation was found. One isolate showed no mutations in PBP3 or *lpoA.* All co-trimoxazole-resistant isolates (15.8%) showed known mutations in *folA* and its promoter region. Additionally, point mutations in *folP* were identified in a subset of these isolates. The most frequent sequence types (STs) were ST57 (*n* = 10) and ST103 (*n* = 10). Genetic cluster analysis identified six clusters, but no epidemiological link could be confirmed.

**Conclusion:**

Resistance to oral antibiotics in non-invasive clinical Hi isolates in Germany was generally low. Amoxicillin is estimated to cover 86% of infections involving non-invasive Hi and, therefore, is still effective for the first-line empirical treatment for ENT infections in Germany. Further surveillance of antimicrobial susceptibility in non-invasive Hi isolates is important to ensure the data basis for guidelines of antibiotic usage.

## Introduction

*Haemophilus influenzae* (Hi) is a Gram-negative coccobacillus and a relevant human pathogen with different manifestations like commensal carriage, non-invasive or invasive disease. In the post-Hib-vaccine era, non-typeable Hi (NTHi) strains evolved to cause the greatest burden of Hi disease, whereby elderly, infants, children and patients with underlying comorbidities have the highest risk of disease.^1^ While the epidemiology of invasive Hi infections is addressed in most research projects,^2,3^ there are few surveillance reports on clinically significant non-invasive diseases due to Hi.

However, non-invasive Hi disease predominantly manifests as infections of mucosal tissue like acute otitis media (AOM), conjunctivitis or exacerbations of chronic pulmonary diseases like COPD. For example, 23% of paediatric bacterial AOM are caused by Hi.^4^ Even though there are restrictive recommendations of antibiotic use for infectious diseases in Germany, ear, nose and throat (ENT) infections are a common cause of antibiotic prescription especially for children.^5^

In Germany, most antibiotics are prescribed in outpatient departments.^6^ The wrong use of antibiotics in hospitals or in outpatient settings results in antimicrobial resistance, which is a severe problem worldwide.^7^ Therefore, the German Paul-Ehrlich-Society for Infection Therapy (PEG) investigates antibiotic resistance rates for several pathogenic microorganisms including Hi in consistent antimicrobial resistance surveillance projects and provides data, which contribute to decisions for guidelines on the empirical usage of antimicrobial drugs in Germany.^8^

The PEG carried out a prospective multicentre surveillance study in 2019/20 and collaborated with the German National Reference Center for Meningococci and Hi (NRZMHi) (1) to provide data on the susceptibility of non-invasive clinical Hi isolates from ENT and respiratory tract infections to oral antibiotics and (2) to study the genetic background of resistances to β-lactam antibiotics, fluoroquinolones, doxycycline, rifampicin and co-trimoxazole.

## Materials and methods

### Isolates

For this prospective study, 215 non-invasive Hi isolates from ENT were collected from a sentinel network of 23 diagnostic laboratories located in five geographic regions across Germany [Table 1; Tables S1 and S2 (available as Supplementary data at *JAC-AMR* Online)]. Each of the diagnostic laboratories was asked to submit 8–10 consecutive, non-duplicate isolates. The period of isolate collection was from October 2019 to March 2020. Two of the isolates could not be recultivated from frozen stocks for serotyping and molecular analyses. The species identification was carried out by MALDI-TOF. In addition, the presence of the Hi*-*specific genes *fucK* and *ompP2/ompP6* (if *fucK* was negative) was confirmed by PCR as described previously.^2^ Serotyping was done by slide agglutination (Remel, Thermo Fisher Scientific, Braunschweig, Germany) and confirmed by PCR of *bexA*. In case of ambiguous results, serotyping was confirmed by serotype-specific PCR.^9,10^

**Table 1.** Specimen of the study isolates and study regions in Germany

Specimen	*n* (%)	Region	*n* (%)
Pharyngeal	68 (31.6%)	North-east	18 (8.4%)
Nose	48 (22.3%)	North-west	26 (12.1%)
Ear	40 (18.6%)	South-east	76 (35.4%)
Eye	28 (13.0%)	South-west	55 (25.6%)
Sputum	22 (10.2%)	West	40 (18.6)
Paranasal sinus	5 (2.3%)		
Other (airways/skin)	4 (1.9%)		
Total			215 (100%)

### Antibiotic susceptibility testing

MICs were determined by broth microdilution as described previously^11^ and interpreted according to the species-specific clinical breakpoints provided by EUCAST, version 13.1.^12^ All isolates were tested by nitrocefin disks (Sigma–Aldrich Chemie GmbH, Taufkirchen, Germany) for the detection of β-lactamases, and subsequently positive isolates were characterized by TEM-1 PCR.^13^

### Genome sequencing

Whole-genome sequencing was performed for all isolates with resistance to at least one antibiotic. DNA was isolated with the Wizard Genomic DNA Purification Kit (Promega, Walldorf, Germany). NextEra XT chemistry (Illumina Inc., San Diego, CA, USA) was used to prepare libraries for a 150-bp paired-end sequencing run on an Illumina NextSeq 500 sequencer. Sequencing was conducted at the core unit SysMed of the University of Wuerzburg. The raw sequence data were assembled by Velvet assembler^14^ integrated in Ridom SeqSphere + software (Ridom GmbH, Münster, Germany).^15^ A previously published core genome multilocus sequence type (cgMLST) scheme was applied with 1178 core genes, created by the cgMLST target definer of the SeqSphere+ software. A minimum spanning tree (MST) was generated based on the cgMLST allelic profiles. Since there is no established allelic difference threshold for MST clusters of Hi cgMLST, an arbitrary threshold was set by six allele differences to define genetic Hi clusters, as has been done previously.^2^ The *ftsI* gene was sequenced in two isolates for which WGS was unsuccessful.^16,17^

### Molecular resistance mechanisms

The genes encoding proteins associated with antimicrobial resistance, i.e. *ftsI*^17^ and *lpoA*^18^ (β-lactams), *gyrA* and *parC*^19,20^ (fluoroquinolones), *rpoB*^21^ (rifampicin) and *folA* and *folP*^22,23^ (co-trimoxazole), were acquired from the genome sequences, translated and aligned with the respective proteins of Hi strain Rd KW20 (accession number L42023.1) as reference^24^ to identify amino acid alterations. PBP3 amino acid substitutions associated with β-lactam resistance were assigned to the established PBP3 group nomenclature.^17^ Known mutations associated with fluoroquinolone resistance were identified in the quinolone resistance-determining regions (QRDRs) of the DNA gyrase and the DNA topoisomerase IV (Table S2).

In addition, mobile resistance genes *bla*, *sul* and *tet* were identified in clean assembled contigs using ResFinder version 4.1.^25–27^

### Statistical analyses

Data processing was done with Excel 365 MSO (Version 2302 Build 16.0.16130.20186, Microsoft, Redmond, WA, USA), and SPSS version 28 (IBM Corp, Armonk, NY, USA) was used for statistical analyses. Available epidemiological data of the isolates comprised age of the patient, sex, site of isolation of the bacterial strain and the collecting centre. To determine the dependencies of these variables on the overall antibiotic resistance and additionally on amoxicillin, cefuroxime and co-trimoxazole resistance, respectively, *χ*^2^ test and, where appropriate, Fisher’s exact test were applied. A 14-year cut-off for children was defined in accordance with the age group definitions of the Robert Koch Institute. Geographical regions of Germany were defined as north-western (Schleswig-Holstein, Hamburg, Bremen and Lower Saxony), north-eastern (Mecklenburg Western Pomerania, Berlin, Brandenburg and Saxony-Anhalt), western (North Rhine-Westphalia), south-western (Hesse, Rhineland-Palatinate, Saarland and Baden-Württemberg) and south-eastern (Bavaria, Saxony and Thuringia). Statistical significance was assumed for *P* < 0.05. The 95% CIs were calculated by binomial method.

## Results

### Characterization of isolates

Isolates were acquired from 23 laboratories located in five regions of Germany. Most isolates were from the south-eastern (35.5%) and the south-western regions (25.6%) whereas only 8% were from the North Eastern region (Table 1).

Sex ratio (female/male) was balanced (0.99). The median age of the patients was 20 years (range: <1–100 years).

Most isolates were acquired from pharyngeal swabs (*n* = 68; 31.6%), nose (*n* = 48; 22.3%) and ear (*n* = 40; 18.6%). Others were obtained from eye (*n* = 28; 13.0%), sputum (*n* = 22; 10.2%), paranasal sinus (*n* = 5; 2.3%), lungs/airways (*n* = 3; 1.4%) and skin (*n* = 1; 0.5%).

Most isolates were NTHi (*n* = 205; 96.2%), and only few were encapsulated, i.e. serotypes e (*n* = 4; 1.9%), f (*n* = 3; 1.4%) and a (*n* = 1; 0.5%). No isolate was serotype b, c or d (Table S1).

### Phenotypic antibiotic susceptibility

All isolates with resistance to at least one antibiotic were NTHi. Over 65% of the isolates were resistant to a single antibiotic with the highest number of resistances observed being five (Table 2).

**Table 2.** MIC distribution for all tested clinical non-invasive Hi isolates

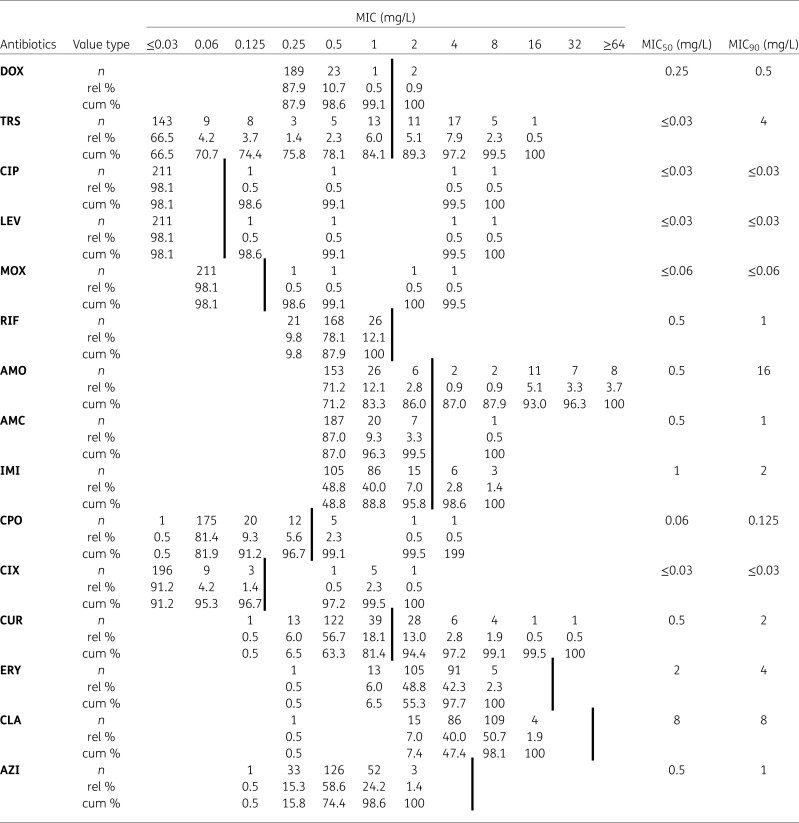

EUCAST breakpoints and ECOFFs for macrolides are indicated by a bold vertical line.

rel %, relative percentage; cum %, cumulative percentage; DOX, doxycycline; TRS, trimethoprim/sulfamethoxazole; CIP, ciprofloxacin; LEV, levofloxacin; MOX, moxifloxacin; RIF, rifampicin; AMO, amoxicillin; AMC, amoxicillin + clavulanic acid; IMI, imipenem; CPO, cefpodoxime; CIX, cefixime; CUR, cefuroxime; AZI, azithromycin; CLA, clarithromycin; ERY, erythromycin.

About 86% (*n* = 185) of the tested isolates were susceptible to amoxicillin. Most of the amoxicillin resistant isolates (*n* = 29; 96.7%) expressed a β-lactamase, one of them was additionally resistant to clavulanic acid. Moreover, one other isolate was amoxicillin resistant, but β-lactamase negative. Among 40 (18.6%) cefuroxime-resistant isolates, only four were also resistant to amoxicillin. All eight isolates resistant to cefpodoxime and/or cefixime were also cefuroxime resistant. Nine (4.2%) isolates were imipenem resistant.

Susceptibility rates were determined for other antibiotic classes, with doxycycline resistance detected in two isolates. All but one isolate was susceptible to rifampicin, and the MIC of the resistant isolate was 2 mg/L, which is close to the breakpoint (*R* > 1 mg/L). Thus, retesting of the isolate by gradient agar diffusion resulted in a susceptible phenotype (MIC 0.5 mg/L). Almost 98% of the isolates were susceptible to ciprofloxacin, moxifloxacin and levofloxacin. Moreover, susceptibility to co-trimoxazole was found in 84% of isolates (Table 2). According to EUCAST criteria for Hi, there are only epidemiological cut-off values (ECOFFs) for macrolides. All MICs of azithromycin, erythromycin und clarithromycin were below the respective ECOFF shown in Table 2.

NTHi (Fisher’s exact test, *P* = 0.025), patients aged >14 years (Pearson’s *χ*^2^ test, *P* = 0.002) and non-ear isolates (Pearson’s *χ*^2^ test, *P* = 0.011) were significantly associated with antibiotic resistance. Amoxicillin resistance was more frequent in isolates from throat (Pearson’s *χ*^2^ test, *P* = 0.027) and nasal swabs (Fisher’s exact test, *P* = 0.031) and from the south-eastern region (Pearson’s *χ*^2^ test, *P* = 0.017). Moreover, cefuroxime resistance was associated with female patients (Pearson’s *χ*^2^ test, *P* = 0.038) and patients older than 14 years (Pearson’s *χ*^2^, *P* < 0.001) and was not linked to the ear (Fisher’s exact test, *P* = 0.045) (Table 3).

**Table 3.** Cross table with *P*-values for all executed *χ*^2^ and Fisher’s exact tests

		Total antibiotic susceptibility		Amoxicillin		Cefuroxime		Co-trimoxazole	
		Resistant (*n* = 82)	Susceptible (*n* = 133)	Resistant (*n* = 36)	Susceptible (*n* = 179)	Resistant (*n* = 40)	Susceptible (*n* = 175)	Resistant (*n* = 34)	Susceptible (*n* = 181)
Age									
	≤14 years (*n* = 103)	28	75	13	90	9	94	12	91
	>14 years (*n* = 112)	54	58	23	89	31	81	22	90
	*P*-value	**0.002**		0.145		**<0.001**		0.135	
Sex									
	Male (*n* = 107)	34	73	17	90	14	93	14	
	Female (*n* = 108)	48	60	19	89	26	82	20	93
	*P*-value	0.056		0.738		**0.038**		0.275	88
Capsulation									
	Capsulated (*n* = 8)	0	8	0	8	0	8	0	8
	NTHi (*n* = 205)	82	123	31	169	40	165	31	171
	*P*-value	**0.025**		0.357		0.357		0.361	
Anatomical Site									
	Pharyngeal (*n* = 68)	32	36	17	51	17	51	12	56
	Else (*n* = 147)	50	97	19	128	23	124	22	125
	*P*-value	0.067		**0.027**		0.101		0.616	
	Ear (*n* = 40)	8	32	3	37	3	37	4	36
	Else (*n* = 175)	74	101	33	142	37	138	30	145
	*P*-value	**0.011**		0.087		**0.045**		0.264	
	Nose (*n* = 47)	16	31	3	44	7	40	7	40
	Else (*n* = 168)	66	102	33	135	33	135	27	141
	*P*-value	0.513		**0.031**		0.460		0.845	
Geographical Site									
	North-east (*n* = 18)	10	8	4	14	37	15	5	13
	Else (*n* = 197)	72	125	32	165	3	160	29	168
	*P*-value	0.102		0.516		1.000		0.173	
	North-west (*n* = 26)	10	16	4	22	6	20	4	22
	Else (*n* = 189)	72	117	32	157	34	155	30	159
	*P*-value	0.731		1.000		0.532		1.000	
	West (*n* = 40)	13	27	4	36	10	30	4	36
	Else (*n* = 175)	69	106	32	143	30	145	30	145
	*P*-value	0.454		0.248		0.249		0.341	
	South-west (*n* = 55)	15	40	5	50	8	47	7	48
	Else (*n* = 160)	67	93	31	129	32	128	27	133
	*P*-value	0.065		0.078		0.370		0.467	
	South-east (*n* = 76)	34	42	19	57	13	63	14	62
	Else (*n* = 139)	48	91	17	122	27	112	20	
	*P*-value	0.114		**0.017**		0.676		0.439	119

*P*-values < 0.05 are presented in bold.

NB: capsulation could only be determined of 213 out of 215 strains.

### Molecular analysis

#### Genetic resistance mechanisms

Molecular resistance patterns could be analysed for 81 of the 82 isolates with phenotypic resistance to at least one antibiotic. All phenotypically β-lactamase-positive amoxicillin-resistant (BLPAR) isolates (*n* = 29) harboured *bla_TEM-1_*. PBP3 mutations were found in one β-lactamase-positive amoxicillin and clavulanic acid-resistant isolate (BLPACR) and one β-lactamase-negative amoxicillin-resistant isolate (BLNAR). Additionally, two phenotypic BLPAR isolates also revealed PBP3 alterations (gBLPACR). Known PBP3 mutations were found in 37 out of 40 cefuroxime-resistant isolates. One of the three remaining cefuroxime-resistant isolates showed the previously described *lpoA* M151I mutation as potential resistance mechanism. Another isolate showed PBP3 mutations D350N, V547I, N569S and E603N, which could not be assigned to a known PBP3 group. No obvious potential resistance mechanism could be detected in this isolate. Furthermore, all isolates resistant to cefpodoxime, cefixime and/or imipenem showed known changes in PBP3 (Table 4).

**Table 4.** Frequency and characteristics of molecular antibiotic resistance markers among identified STs

Antibiotic (number of resistant isolates)	Molecular mechanisms			*n*	ST (*n*/all)
Fluoroquinolones (4)	GyrA		ParC		
	S84L + D88G		S88I	2	Singletons (2)
	S88L		S84I	1	Singleton (1)
	S84L			1	11 (1/2)
Doxycycline (2)	*tet(B)*			2	Singletons (2)
Co-trimoxazole (34)	*folA* promoter	FolA	FolP		
		I95L + F154S/V	P64Ins(SFLYN)+N65D + G189C	4	34 (2/2), Singletons (2)
		I95L	P64Ins(SFLYN)+N65D + G189C	10	57 (2/10), 422 (4/4), 836 (1/3), Singletons (3)
		F154S/V	P64Ins(SFLYN)+N65D + G189C	6	2031 (4/4), Singletons (2)
		F154S	N65D + G189C	1	Singleton (1)
		I95L	G189C	1	836 (1/3)
		I95L + F154V	*—*	6	57 (6/10)
		F154S	*—*	2	Singletons (2)
		I95L	*—*	2	Singletons (2)
		FolA (F154S) + *sul2*		1	Singleton (1)
	n.d.			1	n.d.
β-Lactams BLNAR (g)BLPACR Imipenem Cefuroxime (41)	PBP3 Group I			1	Singleton (1)
	PBP3 Group IIa			8	57 (1/10), 425 (1/2), Singletons (6)
	PBP3 Group IIb			13	411 (2/2), 422 (3/4), Singletons (7), n.d. (1)
	PBP3 Group IIc			4	11 (1/2), 57 (1/10), Singletons (2)
	PBP3 Group IId			6	11 (1/2), 57 (1/10), 425 (1/2), 1202 (2/2), Singleton (1)
	PBP3 Group III			1	2031 (1/4)
	PBP3 Group III+			1	Singleton (1)
	PBP3 Group III like			3	2031 (3/4)
	PBP3 Group III like+			1	103 (1/10)
	Penicillin-binding protein activator mutation			1	Singleton (1)
	Other			2	Singletons (2)
BLPAR BLPACR (29)	*bla*TEM-1			29	57 (2/10), 103 (10/10), 160 (2/2), 388 (2/2), 836 (3/3), 2031 (1/4), 2596 (2/2) Singletons (6), n.d. (1)

BLNAR, β-lactamase-negative ampicillin resistance; (g)BLPACR, (genetic) β-lactamase-positive amoxicillin clavulanic acid resistance; BLPAR, β-lactamase-positive ampicillin resistance.

All 33 genome-sequenced co-trimoxazole-resistant isolates harboured the known mutation I95L or F154S/V or both in FolA. The P64Ins(SFLYN), N65D and G189C mutations in FolP were present in 20 resistant isolates (58.8%). In addition, these FolP mutations were partially found in two other isolates. The mobile *sul2* gene encoding a dihydropteroate synthase was detected in one isolate. Moreover, all four fluoroquinolone-resistant isolates showed amino acid substitutions in the QRDRs of GyrA. The S84I ParC alteration was found in three isolates. The two doxycycline-resistant isolates possessed the transferable *tet(B)* gene, which encodes a tetracycline efflux pump (Table 4).

### Genetic diversity

**Figure 1.**
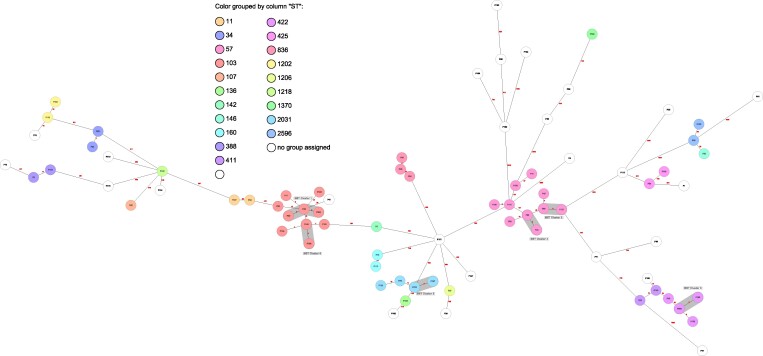
MST based on cgMLST data of 80 whole-genome sequences from Hi isolates created with Ridom SeqSphere+ software. Six allele differences were set as cluster distance threshold and consequent clusters were highlighted in grey. Colours indicate the conventional MLST based on seven housekeeping genes.

Phylogenetic analysis by cgMLST included 80 isolates with resistance to at least one antibiotic. In total, the isolates were assigned to 46 different sequence types (STs), of which four were new (STs 2894–2897), and 23 clonal complexes. Most frequent STs were ST57 (*n* = 10), ST103 (*n* = 10), ST422 (*n* = 4), ST2031 (*n* = 4) and ST836 (*n* = 3). All other STs occurred once or twice (Table S1). By setting an arbitrary cluster distance threshold at six allele differences, six genetic clusters were identified, one comprising three isolates, whereas the remaining clusters consisted of two isolates (Figure 1). Overall, four out of six clusters exhibited identical phenotypic and genotypic resistance patterns.

## Discussion

To our knowledge, this is the first study providing an insight into the antibiotic resistance and the molecular epidemiology of Hi isolates from non-invasive ENT infections in Germany. The prevalence of antibiotic resistance in Germany has been already thoroughly studied for invasive isolates.^2,3,28^ This study complements the previous research by providing important data on non-invasive isolates concerning oral antibiotics.

Overall, low resistance rates were found for all tested antibiotics. In comparison with a similar study conducted in 2013,^11^ only minor increases of resistance rates were detected for all antibiotics: resistances to amoxicillin [14.0% (95% CI 9.8–19.1%) versus 10.3% (95% CI 6.5–15.3%) 2013], amoxicillin + clavulanic acid [0.5% (95% CI 0.1–2.2%) versus 0% (95% CI 0–1.3%) 2013], cefuroxime [18.6% (95% CI 13.8–24.2%) versus 13.5% (95% CI 9.2–19.0%) 2013], cefixime [3.3% (95% CI 1.5–6.3%) versus 1.1% (95% CI 0.2–3.4%) 2013], co-trimoxazole [15.8% (95% CI 11.4–21.1%) versus 9.7% (95% CI 6.1–14.6%) 2013], ciprofloxacin [1.9% (95% CI 0.6–4.4%) versus 0.5% (95% CI 0.1–2.5%) 2013] and doxycycline [0.9% (95% CI 0.2–3.0%) versus 0% (95% CI 0–1.3%) 2013] were insignificantly higher. Only the cefpodoxime resistance rate was significantly increased [3.3% (95% CI 1.5–6.3%] versus 0% (95% CI 0–1.3%) 2013].

A recent survey from France reported much higher resistance rates for β-lactam antibiotics (amoxicillin: 61.4%; cefotaxime: 39.3%) and co-trimoxazole (33.2%) in non-invasive isolates, while susceptibility rates detected for ciprofloxacin (91.8%) and rifampicin (99.7%) were comparable with our data.^29^ Previously published data on invasive Hi isolates from Germany have shown a significant increase of resistance to aminopenicillins.^30,31^ Although this trend is not seen in our data for non-invasive Hi, it remains important to prevent increasing resistance rates by sufficient empirical treatment.

Recommendations regarding thresholds of antibiotic resistance rates at which empirical therapy is no longer advised vary and depend on different factors.^32^ Given that non-invasive infections are not life-threatening, our data support an empirical therapy with aminopenicillins for ENT infections, which would be effective for approximately 86% of cases involving Hi. Moreover, considering significant associations between higher antibiotic susceptibility rates and younger patients, serotypes and anatomical or geographical sites of isolation could be useful for novel recommendations of empirical antibiotic usage.

Our finding that antibiotic resistance was inversely correlated with specimens from the ear may reflect the common guidelines recommending that otitis media should primarily not be treated with antibiotics.^33^ Otitis media is a prevalent condition, especially in paediatric patients, and is often caused by Hi. However, it is now widely accepted that otitis media is self-limiting. The reduced use of antibiotics for this condition may have contributed to the observed decrease in resistance rates among Hi isolates from the ear.

The data of our study showed that non-invasive Hi was significantly less resistant in patients of younger age groups. The fact that invasive Hi infections are conditions of the elderly in Germany^31^ may explain the observed lower resistance rate in non-invasive strains compared with isolates from invasive disease. It is conceivable that Hi isolates become more resistant as a patient gets increasingly exposed to antibiotics with age.

The most prevalent mechanism of amoxicillin resistance was the presence of a β-lactamase (*bla_TEM-1_*) as noted in the previous study on non-invasive Hi^11^ and in a global antibiotic susceptibility survey.^34^ In contrast to the previous data on ENT-causing Hi isolates, low rates of BLNAR and BLPACR strains were identified in the present study. Even though this concerned only few isolates, the emergence of PBP3-mutated strains points at a trend towards resistance to β-lactam antibiotics even in non-invasive Hi. PBP3 Group IIa and Group IIb defining amino acid substitutions were found as the most frequent molecular mechanisms of cephalosporins and imipenem resistance.^35,36^ We did not find resistance-associated PBP3 alterations in three cefuroxime-resistant isolates, while one of them exhibited a *lpoA* mutation as a potential resistance mechanism.^18^ It is noteworthy that only few cefuroxime-resistant strains were amoxicillin resistant. For all co-trimoxazole-resistant isolates, molecular resistance determinants were found. Alterations in the *folA* promoter as well as *folA* and *folP* mutations associated with resistance to trimethoprim were prevalent in all co-trimoxazole-resistant isolates analysed. Sulfamethoxazole resistance markers *folP* and *sul2* were found less frequently. This corresponds to previous reports describing FolA alterations as the main cause of co-trimoxazole resistance in Hi while substitutions in FolP and the presence of *sul2* seem to contribute additionally to higher MIC values.^37^ Known *parC* or *gyrA* mutations were detected in all phenotypically ciprofloxacin-resistant isolates with the highest MICs in isolates with two GyrA mutations (S84L and D88G). The doxycycline-resistant isolates exhibited *tet(B)*, which was also observed in a recent study from Norway.^38^

cgMLST revealed high genetic diversity between antimicrobial resistant Hi isolates, as described in other studies worldwide.^39–41^ Consistent with findings from other studies,^42^ all STs (except the newly identified) have also been detected in invasive Hi isolates in Germany (Frank *et al.*; unpublished data). This suggests that non-invasive isolates do not belong to different populations than invasive isolates and supports the common concept that invasive infections as well as non-invasive infections arise from carriage.^43–45^ ST103, an internationally prevalent ST known for its association with β-lactamase producers,^38,40^ was among the most frequently identified in this study, with *bla_TEM-1_* detected in all isolates. Isolates of ST57, previously reported in both carriage studies^46^ and studies of invasive isolates,^39^ demonstrated variable molecular resistance patterns. Skaare *et al.*^47^ and Tsang *et al.*^41^ reported that PBP3 groups are possibly linked to specific STs. In this study, ST411 and ST1202 isolates were classified within PBP3 Groups IIb and IId, while all ST2031 isolates were within PBP3 Groups III and III like. Furthermore, FolA/FolP alteration patterns were observed to be similar within ST34, ST422 and ST2031. The identified clusters of ST103 and ST57 exhibited identical phenotypic and genotypic resistance patterns, whereas the clusters of ST2031 and ST422 showed genotypic and or phenotypic variations. In spite of these common features, clustering isolates were collected from different laboratories and no epidemiological data were available.

This study has several limitations: The study was designed as a cross-sectional *in vitro* resistance surveillance of non-invasive Hi isolates. However, the clinical relevance of the included strains was determined based only on the assessment of the submitting clinicians. Therefore, the inclusion of the study isolates was not fully objective. The conducted analyses of molecular resistance mechanisms are not comprehensive and serve as preliminary assessment. Further research is needed on the resistance mechanism in Hi. In particular, the role of PBP3 mutations for the resistance to β-lactam antibiotics remains an open question. Additionally, the establishment of the cluster distance threshold in our phylogenetic analysis was arbitrary. An epidemiologic validation was beyond the scope of this study. Although genetic clusters displaying differences in resistance patterns were identified, it remains uncertain whether the arbitrarily set threshold was sufficiently sensitive.

In conclusion, the high antibiotic susceptibility rates observed in this study support the recommendation of amoxicillin as the first-line empirical treatment for ENT infections in Germany. Based on our data, this treatment would effectively cover approximately 86% of Hi infections. Nevertheless, given the significant risk of antibiotic resistance development, cautious antibiotic prescribing is essential. The identification of internationally spreading resistant NTHi, such as ST103, underlined the need for global molecular surveillance. Continued evaluation of antibiotic resistance rates, along with their combined phenotypic and genotypic characterization, is crucial for monitoring emerging trends in antibiotic resistance and for providing accurate treatment recommendations.

## Supplementary Material

dlae197_Supplementary_Data

## Supplementary data

Tables S1 and S2 are available as Supplementary data at *JAC-AMR* Online.
