# Supplementary material for: Antibiotic resistance and molecular characterization of non-invasive clinical Haemophilus influenzae isolates in Germany 2019 and 2020
Source: JAC Antimicrob Resist. 2024 Dec 10;6(6):dlae197. doi: 10.1093/jacamr/dlae197 (PMC11631347; doi:10.1093/jacamr/dlae197)
Supplement: dlae197_Supplementary_Data [file dlae197_supplementary_data.zip › Supplementary Table S2.docx]

Supplementary Table S2

| Antibiotic | Gene | Protein | AA mutations | References  (cf. main manuscript) |
| --- | --- | --- | --- | --- |
| β-lactams | *ftsI* | PBP3 |  | ^2, 17^ |
|  |  | I | R517H |  |
|  |  | IIa | N526K |  |
|  |  | IIb | N526K + A502V |  |
|  |  | IIc | N526K +A502T |  |
|  |  | IId | N526K + I449V |  |
|  |  | III | M377I + S385T + N526K |  |
|  |  | III-like | M377I + S385T + R517H |  |
|  |  | III+ | M377I + S385T + L389F + N526K |  |
|  |  | III-like+ | M377I + S385T + L389F + R517H |  |
|  | *lpoA* | PBP activator | M151I | ^18^ |
| Ciprofloxacin | *gyrA* | DNA gyrase | S84L, D88G | ^19, 20^ |
|  | *parC* | DNA topoisomerase IV | S84I |  |
| Cotrimoxazole | *folA* | Dihydrofolate reduktase | folA promotor changes; I95L; F154S/V | ^22, 23^ |
|  | *folP* | Dihydropteroate synthase | P64Ins(SFLYN), N65D, G189C |  |
| Rifampicin | *rpoB* | RNA polymerase β-subunit | Q513L, D516A/V/N/Y, N518D, H526L, L533S | ^21^ |

Supplementary Table S2 Previously described significant mutations in antibiotic resistance related genes analyzed in this study.
